# Biological information specialists for biological informatics

**DOI:** 10.1186/1747-5333-2-1

**Published:** 2007-02-12

**Authors:** P Bryan Heidorn, Carole L Palmer, Dan Wright

**Affiliations:** 1 Graduate School of Library and Information Science, University of Illinois at Urbana-Champaign, USA

## Abstract

Data management and integration are complicated and ongoing problems that will require commitment of resources and expertise from the various biological science communities. Primary components of successful cross-scale integration are smooth information management and migration from one context to another. We call for a broadening of the definition of bioinformatics and bioinformatics training to span biological disciplines and biological scales. Training programs are needed that educate a new kind of informatics professional, Biological Information Specialists, to work in collaboration with various discipline-specific research personnel. Biological Information Specialists are an extension of the informationist movement that began within library and information science (LIS) over 30 years ago as a professional position to fill a gap in clinical medicine. These professionals will help advance science by improving access to scientific information and by freeing scientists who are not interested in data management to concentrate on their science.

## Background

There is a growing awareness of the need to work toward the integration of data across biological scales, from the biomolecular to ecosystems. In particular, recent reports on cyberinfrastructure and e-science initiatives recognize the shortage in qualified professionals to manage the increasing stores of scientific data [1]. Data management and integration are complicated and ongoing problems that will require commitment of resources and expertise from the various biological science communities. Data issues include, for example, formal standards-based representation of experimental conditions, procedures, and generated data to allow for data federation and so that unique applications do not need to be built for each data set. And, while data issues are central to the future of the scientific enterprise, they do not exist in isolation. They are part of a larger family of information and communication activities that have emerged from the swift development of many new and essential technologies across the biological domains. Broad changes and advancements in information use are impacting all modes of scientific inquiry, from the administration of big science to the conduct of daily bench work, in all fields of biological research.

As part of this trend, bioinformatics programs are being developed across the country. They focus effectively on issues such as molecular modeling and gene ontologies; however, with the exception of some medical informatics programs, they do not cover in a comprehensive way the broad range of biological information concerns including data exchange standards, digital preservation, and electronic publishing. Bioinformatics programs at universities tend to focus on computational molecular biology [2], though bioinformatics has been broadly construed in segments of the scientific community as applying to all scales of biological data, as evidenced in the NIH Biomedical Information Science and Technology Initiative (BISTI) documentation: "Research, development, or application of computational tools and approaches for expanding the use of biological, medical, behavioral or health data, including those to acquire, store, organize, archive, analyze, or visualize such data" [3].

Existing educational programs have tended to concentrate on the analysis, computation and visualization of molecular data or health information. In biomedical informatics, there are a number of programs in informatics broadly defined [[4], p. *xv*]. These programs tend to define informaticists as operating in the clinical setting. For example, the Medical Library Association Report following an Annals of Internal Medicine editorial [5] "An informaticist ..., possesses in-depth knowledge in both clinical medicine and information seeking and appraisal and employs that knowledge as part of a clinical team" [6]. In this paper we advocate taking the lessons learned and questions posed in *clinical informaticist *training programs and practice and applying them to the broader definition of *biological informatics*. There is a need to educate a new generation of information specialists who are skilled in the many aspects of information management and integration across scale and across fields of biology. This should be done for good reason – scientists need to focus their efforts on conducting science, not managing information or struggling to develop, use, and maintain their information systems.

Integration of data and results across scales will only be attainable if the range of biological sciences is orchestrated in this effort. At the same time, information systems for scientists need to be grounded in a deep understanding of distinct research interests and activities of different biological domains [7,8]. In recognition of the long-term aims of broad trans-scale integration in science [9], we conceive of this information science-based initiative in bioinformatics as "biological informatics." In direct response to the qualitative changes in biological research and specific workforce gaps, we are developing a biological informatics masters degree program to train a new generation of information science professionals. These biological information specialists will be trained to support research and communication in local scientific research environments while also working more globally to develop shared approaches to long-lived data and integration of information and tools across biology.

## Biological Informatics

The relationship between "bioinformatics" and "biological informatics" is not as subtle as it might seem. Over the past ten years in the U.S., the term "bioinformatics" has generally been used to mean "information about molecular biology", particularly gene and protein sequences. This use of the term in the popular press, associated with the great progress and success in that field, has served to cement this definition into the psyches of the general population and scientists alike – thus the need for a new term (biological informatics) to cover the science of information about all levels of biological analysis. Health informatics, medical informatics, neuroinformatics, as well as biodiversity informatics and biomolecular informatics, all fall under this broader concept [10,11]. While we use the term "biological informatics" here to clarify the breadth of the concept, there is good reason to argue for reclaiming the name "bioinformatics" to cover all information about biology. For example, biodiversity and ecological informatics, fields often overlooked in discussions of bioinformatics, are an essential component of our conception of biological informatics. For more on this question, please see the comments by Hersh and our response to him, which can be found in the Readers' Comments section accompanying this article.

Biodiversity informatics is the study of data problems where information acquisition, analysis, sharing, and collaboration are required to answer broad questions about biodiversity. Biological diversity means "the variability among living organisms from all sources, including *inter alia*, terrestrial, marine and other aquatic ecosystems and the ecological complexes of which they are part; this includes diversity within species, between species and of ecosystems" [12]. Informatics is as vital to biodiversity biologists as it is to molecular biologists. As E. O. Wilson states, biologists are turning to information technology to produce critically needed efficiencies in their work, but much more effort is needed: "New electronic technology, increasing exponentially in power, is trimming the cost and time required for taxonomic description and data analysis. It promises to speed traditional systematics by 2 orders of magnitude. What is lacking and needed now is a concerted effort, comparable to the Human Genome Project (HGP), to complete a global biodiversity survey – pole to pole, whales to bacteria, and in a reasonably short period of time" [13].  Changes in information technology have affected the face of biodiversity on the local level for scientists, but the field has undergone a revolutionary globalization and shift in scale which has introduced new challenges for biological informatics.

Biodiversity informatics overlaps with other branches of biology such as medical informatics and public health informatics in areas such as those related to disease vector (i.e. West Nile Virus mosquito vectors) natural history and climate driven species distribution changes, yet there are few individuals trained to cross the boundaries between fields such as entomology, botany or zoology. This overlap produces a demand for shared electronic data. It is well recognized that long-distance collaboration and data sharing is "good" for science, and a number of projects exist that demonstrate the commonality of problems and goals in this area, across a range of biological disciplines.

An important commonality among large-scale biological informatics projects is the need for efficient storage of large volumes of data and for standardized formats that facilitate access by the wider scientific community. The Protein Data Bank (PDB), the Global Biodiversity Information Facility (GBIF), and the Biomedical Informatics Research Network (BIRN) have all recognized this challenge and are meeting it in several different ways. Data quality and deposition standards are addressed in each project's specific solution to the problems of data storage and access. Another challenge in the use and administration of data resources is coordination between the researchers, journals, and the repository itself to ensure timely and useful availability of data. Again, it is instructive that in confronting this problem each of the aforementioned projects has taken a different but related approach stemming from the specific requirements of each subdiscipline's data.

The need for integration across fields and the commonalities underlying the problems confronted by different biological informatics projects, we believe, calls for broadly trained informatics professionals with a strong base of biological knowledge. These biological information specialists (BISs) will work in collaboration with various discipline-specific research personnel in the biosciences to solve the problems associated with the overarching information deluge in the biological sciences.

## Biological Information Specialists

Our conception of the BIS is an extension of the informationist movement that began within library and information science (LIS) over 30 years ago. Beginning with an emphasis on clinical medical librarianship, informationists have now advanced beyond the clinical realm to also work as members of scientific research groups toward similar goals of improving information use and communication among teams. Clearly, some of the same technological and social forces that have molded the medical informationist movement are now impacting all of the biological sciences.

Despite the very real contributions informationists have made to practicing clinical medicine, a disconnect remains between biomedical research, clinical practice, and health care provision. Moreover, new complexities have been introduced with information technology and the pervasiveness of concepts like evidence-based medicine, which explicitly call for the integration of research evidence with patient care, yet the knowledge that resides in medical and health-related journals, databases, and other resources often goes unused. The medical informaticists and the clinical librarian tend to be closely linked to one another and to access to the biomedical literature [14,5]. The same is true for non-medical informaticists and for biology librarians. However, the nature of publishing and scientific discovery are changing. Increasingly, primary and secondary data are becoming auxiliary parts of publications or publication on their own right. Therefore, BISs will need to be competent with literature organization and searching but also with direct management of the primary data being generated by scientists and increasingly shared among distributed groups of scientists. The BIS information science skills will facilitate both the consumption and production of research information.

Informationists, working in collaboration with teams of medical scientists to facilitate their interaction with and use of information resources, may come from either information or health-related backgrounds [15]. And, as has been the experience in *clinical informatics*, in some cases the individuals entering the BIS program will have prior training in either biology or information science at the undergraduate, masters, or the doctoral level. But in general, as Florance et al. [16] explain, preparing information specialists to work in "information-rich environments and to participate as peers in problem solving" *requires cross training in library and information science and disciplinary knowledge in scientific domains*. Moreover, their training should include an internship in a practice setting. In the contemporary biological research environment BISs will need a balance of skills that spans the scientific research domain and information science, as well as a practical understanding of the biological research process. Our experience to date also indicates that some graduate degree candidates in the biological sciences may turn to our program to augment their education with informatics training to gain advantage in highly competitive branches of biology.

At present, there are more information resources available to biological researchers, from systematists to physicians, than ever before, and countless more are in development. They range from bibliographic and textual information to raw data, and include Internet websites, data analysis software, visualization tools, and databases of published literature, DNA and protein sequences, and various kinds of image data. LIS is a vital contributor to the management, integration, and use of information resources, because it is the only field that is concerned with the full landscape of scientific information and the interactions therein, and with the provision of services to exploit that base of knowledge [17,18]. BISs will have appropriate training to marshal that knowledge to solve information problems in concert with scientists, while complementing, not duplicating, the expertise of computational scientists. Computational scientists will continue to be essential to advancing the state-of-the-art in computational biology. BISs will be central in developing the cyberinfrastructure and information services necessary to facilitate interdisciplinary and multi-scale science – aspects of scientific work that the NSF and NIH have identified as key to the future conduct of research.

The BIS skill set will focus specifically on the following areas:

1) Evaluation and implementation of information systems: user based assessment and continual quality improvement for the development of tools that work and are used.

2) Information acquisition, management, and dissemination: development of digital libraries, data archives, institutional repositories, and related tools (e.g. data curation).

3) Information organization and integration: ontology development, structuring information for optimal use and sharing, and standards development.

Within the curriculum, these skills will be mapped to coursework, internships, and thesis work (see the degree requirements at [18]). Requirements include coursework in four core areas: biology, bioinformatics, computer science, and information science.

Part of our program is to track employment trends and opportunities for BISs and use that information to inform our continued curriculum development. We expect there will also be a need to build awareness among scientists, large research labs, and funding agencies about the value of BISs for increasing scientific production. And, while some labs are too small to be able to afford dedicated professionals, BIS support services can be centralized to spread costs over many projects and units at an institution, as is the current model with research libraries. The BIS training will be applicable to the range of scientific research environments.

## Contribution to Science

BIS graduates will contribute to science by making information more useful to more scientists. They will also free scientists who are not primarily interested in data management to concentrate on research. The problems of scientific information management and integration are acute and are escalating each day. National and international funding bodies increasingly see support for research projects not only as investments in the publication of research findings in journal articles, because these publications are no longer seen as the sole final product of the scientific enterprise. The agencies now recognize that they are also investing in the creation of data repositories that can serve as the raw material for future science. Scientists will need to begin to treat data in ways that are fundamentally different from the practices of the past. Collection and storage of data will require consideration of future interoperability and usefulness in other wide-ranging contexts, not just the applications of a single experiment or a particular lab. This necessarily involves many information management techniques and practices that are beyond the scope of what biological scientists are accustomed to, and that would be burdensome on top of the rigors of the everyday conduct of science. However, for scientists who are interested or must interact personally with complex information technology the BIS can play an instructional role as well as that of intermediary.

The BIS program aims to train professionals to provide support throughout the biological sciences. This cross-disciplinary approach has a number of advantages, primarily that solutions found by BISs working with scientists in one particular discipline can be applied to data problems in other disciplines. Examples of problems that are universal across disciplines include: data federation, API development, data storage formats, and archiving. However, unlike many information technology jobs, BIS work will require significant knowledge of the biological domains served. Students will not only gain a broad understanding of scientific communication and information organization, retrieval, and management, they will also be required to develop a strong understanding of how informatics fits within the biological sciences.

To this end, it is essential that practicing research scientists guide how these professionals will be trained. We have received funding from NSF to partner with scientists from several disciplines of biology from several institutions, and intend to expand participation over the next few years. Our current partners include representatives from the Smithsonian Institution, Missouri Botanical Garden, the Department of Psychiatry at the University of Illinois at Chicago, and the Biomedical Informatics Research Network (BIRN). They are participating as part of our scientific advisory board, as internship supervisors, and as visiting lecturers. We are actively working to recruit additional advisors and collaborators to assist in defining best practices and overarching principles in biological informatics. Through these collaborations we also expect to begin new and useful research projects, which would be difficult without the interaction related to teaching and internships, and continue our work toward expanding our understanding of the role of informatics in scientific progress (see, for example, [10,11,19]).

## Conclusion

Leading researchers in biology have recognized emergent complexity and cross-scale phenomenon to be forces driving the future of the biological sciences [20]. In addition, the National Science Foundation has identified integration across scale as one of the fundamental challenges facing science in the 21^st ^century. A primary component of successful cross-scale integration is smooth information management and migration from one context to another. When integrated into scientific laboratories, BISs will enable the success of this kind of science. By training experts to handle information management and integration tasks, we hope to allow biological scientists to concentrate on doing science and improve the quality and portability of scientific information. Ultimately BISs will be able to fill a new but essential role in biological science research settings, resulting in better biological science *and *better information science.
